# Tail-like anther crest aids pollination by manipulating pollinator’s behaviour in a wild ginger

**DOI:** 10.1038/srep22340

**Published:** 2016-03-01

**Authors:** Yong-Li Fan, Qing-Jun Li

**Affiliations:** 1 Key Laboratory of Tropical Forest Ecology, Xishuangbanna Tropical Botanical Garden, the Chinese Academy of Sciences, 88# Xuefu Road, Yunnan 650223, China; 2 China Forest Exploration and Design Institute of Kunming, Kunming 650216, China

## Abstract

Innovative floral organs are widely distributed taxonomically in angiosperms, and some of them are conspicuous and curious in morphology. Floral organs have long been supposed to play a crucial role in fertilization by pollinators. However, why innovative organs occur, how they are adapted for pollinators and what sexual roles they play are still puzzling. Here we focused on a wild ginger (*Zingiber densissimum*, Zingiberaceae) and tested the function of the curious anther crest, an innovative floral structure widely distributed in Zingiberaceae. The anther crest is a specialized anther appendage that extends up from the top of the anther to form a tail-like structure, about 150% as long as the anther. We found this structure promoted both the male and the female functions of plants by manipulating its pollinators and causing pollinators to adopt a position ideal for pollen removal and receipt. This study provides a novel example of structure adaptation in which both the male and the female functions are enhanced by resource allocation on a male organ, expanding the knowledge of the sexual roles of the anther appendage.

Since Darwin, it has been interpreted that flowers are adapted for fertilization by pollinators^1^. Floral traits, such as color, shape and size, can be fully understood only when considering their functions in adaptation for pollinators^2^,^3^, since floral traits are primarily shaped during the mutual selection between flowers and their pollinators^1^,^4^,^5^,^6^.

In angiosperms, innovative floral organs, such as vestigial and derived organs, are widely distributed taxonomically, occurring at least in 32.5% of families and 54.4% of genera in these families^7^, with some innovative floral organs strange and conspicuous in morphology. Why these organs occur and how they are adapted for pollinators have long been puzzling^8^,^9^. Limited evidences show that the innovative organ plays a key role in pollination process. For example, the curious sterile inflorescence axis of the South African Cape endemic plant (*Babiana ringens*, Iridaceae) functions to provide a perch for its bird pollinators and cause its pollinator to adopt a position ideal for the cross-pollination of its ground-level flowers, thus promoting seed production^10^. The anther appendage in numerous plant groups, such as genera of *Salvia* (Lamiaceae), *Torenia* (Scrophulariaceae) and *Incarvillea* (Bignoniaceae), functions to promote pollen dispersal via different manners^11^,^12^,^13^. Nevertheless, previous studies on innovative organs were limited to test the function either on seed production (female function) or on pollen dispersal (male function). Few studies have simultaneously examined the bisexual roles, and even no studies link the functions of innovative organs to resource allocation on two genders.

Zingiberales, ancestrally pollinated by animals, have exhibited extremely diversified pollination syndromes, including the syndromes of bees, moths, butterflies, flies, beetles, birds, bats and mammals (lemurs) — almost all of the biotic pollination syndromes recorded in angiosperms^14^,^15^,^16^. This plant group therefore provides an ideal model to explore natural selection and coevolution between plants and pollinators. Flowers in more than 25 genera of ginger family (Zingiberaceae) have an innovative floral structure — anther crest, a specialized anther appendage which extends up from the top of the anther. With great variation in shape and size among different genera, this derived structure is widely distributed in Zingiberaceae. However, it is still unknown why such a device is kept during natural selection. Here we focus on the curious anther crest of a wild ginger (*Zingiber densissimum*). About 150% as long as the anther, the specialized yellow anther crest in this species elongates from the top of the anther and tapers off to a point, forming a tail-like anther crest (TAC) (Fig. 1A,B). Such a conspicuous anther crest is extremely rare even in the entire angiosperm flowers. We are therefore intrigued to explore why flowers invest such considerable resource on this contrivance. We test several adaptive possibilities in sexual reproduction as follows. (1) Does the TAC function as a resource pool to promote seed development after fertilization of ovules? (2) Is the TAC attractive to pollinators during pollination process? (3) Does the TAC have the function of manipulating pollinators’ foraging behaviours? We then proceed to evaluate the contributions of the TAC on the male and the female fitness of plants, respectively, and examine whether the functional role of this contrivance in pollination process matches its sexual role on resource allocation.

## Results

### Functions of the TAC

The TAC-removed and hand self-pollinated flowers produced 11.4 ± 1.2 seeds (n = 31), as many as the intact and hand self-pollinated flowers that produced 12.1 ± 0.9 seeds (n = 36) (t-test, *t* = 0.47, *P* = 0.64). This indicates that TAC removal does not affect seed provisioning.

The intact flower got first visited in 29 floral pairs, while the TAC-removed flower got first visited in 21 floral pairs. There is no significant difference between the intact flowers and the TAC-removed flowers on pollinator attraction (df = 1, *χ*^*2*^ = 1.96, *P* = 0.1615). This result indicates that the TAC has little function on pollinator attraction.

**Figure 1:**
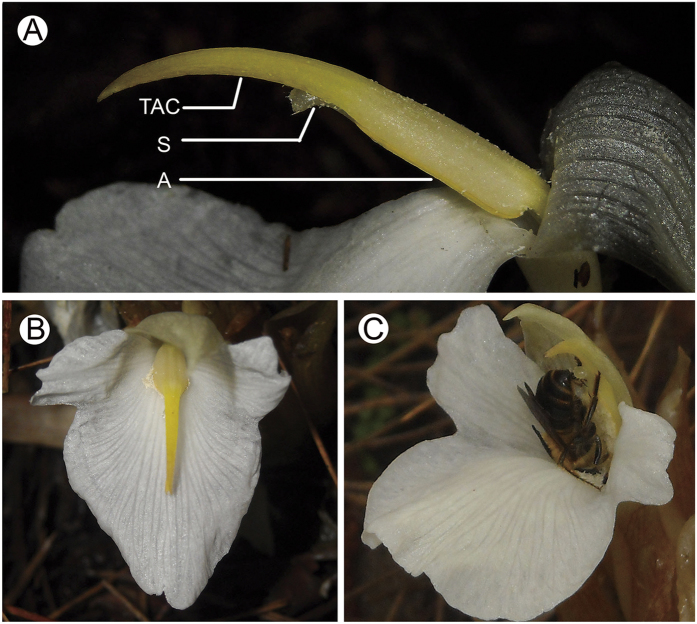
Floral structure and pollinator’s behaviour. (**A**) The inner structure of *Zingiber densissimum* flower: TAC, tail-like anther crest; S, stigma; A, anther. (**B**) Vertical view of a *Z. densissimum* flower, showing the yellow anther crest on the silvery white background of the labellum. (**C**) A bee (*Amegilla zonata*) is visiting a flower in upright-down manner, with its legs holding the anther crest. All the images in this figure were taken by author Yong-Li Fan.

**Figure 2:**
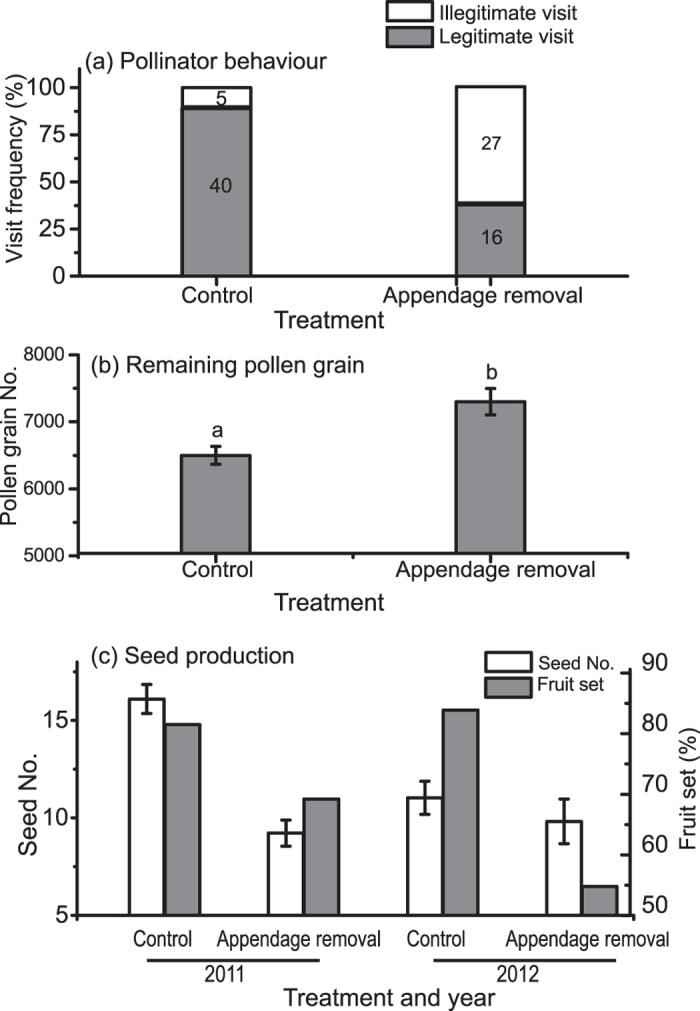
The function of the tail-like anther crest on reproductive success. (**a**) The function of the tail-like anther crest on pollinator’s behaviour, values in the histogram show the number of visit by pollinators. (**b**) The function of the tail-like anther crest on pollen removal, values with different letters indicate the difference is significant. The effect of pollinator species is not shown either in (**a**) or in (**b**), as the composition of the two bee species was not significantly different between the control and the manipulation group (see Table 1). (**c**) The function of the tail-like anther crest on seed production.

Two bee species, *Macropis hedini* (Melittidae) and *Amegilla zonata* (Apidae), were the pollinators of *Z. densissimum*^17^. During nectar foraging, *M. hedini* generally visited flowers in upright manner and pushed up the TAC with its head, achieving dorsal pollination, while *A. zonata* probed nectar in upright-down manner and pushed up the TAC with its legs, achieving ventral pollination (legitimate visit) (Fig. 1C, Supplementary video 1). The TAC acted as a handle for pollinators to push up the anther during their foraging process (Fig. 1C, Supplementary video 1)^17^. In TAC-removed flowers, significantly more pollinator individuals of the two pollinator species did not push up the anther during nectar foraging (Fig. 2A, Table 1). Instead, they directly inserted the proboscis into the floral tube from the side of the filament, without touching the stigma or the anther (illegitimate visit) (Supplementary video 2). This reveals that the TAC functions to manipulate pollinator’s foraging behaviour.

### Evaluation on sexual roles of the TAC

After a single visit by pollinator, pollen grains left on the control flowers were significantly fewer than those left on the TAC-removed flowers (Fig. 2B, Table 1), indicating the TAC promotes pollen removal. The contribution of the anther crest on male fitness (*C*_*m*_) was 40.7%. Flowers in control group had significantly higher fruit set (Fig. 2C, Table 2), and produced significantly more seeds than flowers in manipulation group (Fig. 2C, Table 2), showing the TAC functions to promote pollen deposition. The contribution of the anther crest on female fitness (*C*_*f*_) was 51.4% in 2011, and 50.5% in 2012. These results indicate that TAC functions as both male and female during pollination success of plants, with female function even greater than male.

## Discussion

Our study shows that the TAC plays a key role on pollinator’s behaviour manipulation, while it has little function on pollinator attraction or on seed development. To achieve pollination success, flowers provide diverse rewards and reward signals to attract pollinators^18^,^19^,^20^,^21^. However, upon attracted, pollinators are coming for getting rewards rather than providing pollination service for flowers^22^,^23^. Thereby, in addition to attract pollinators, flowers should develop other strategies or structures to ensure precise pollination by pollinators. The style of flowers in Zingiberaceae is generally soft and string-like, and fixed by the anther (Fig. 1A). The whole anther with the TAC is positioned in the entrance of the corolla tube, an obstacle for pollinators to get the nectar. Therefore, pushing up the anther by pollinators is an essential process for pollen dispersal and deposition. In intact flower group, majority of bees (about 90%) pushed up the anther during nectar foraging, acting primarily as the pollinator. The TAC provides bees with a handle during their nectar foraging (Fig. 2A, Table 1, Supplementary video 1). By contrast, in TAC-removed flower group, significantly more bees directly probed the nectar from the side of the filament in illegitimate manner without pushing up the anther, acting more as the nectar thief than the pollinator (Fig. 2A, Table 1, Supplementary video 2). The results indicate the TAC functions to manipulate pollinator’s behaviour and cause pollinators to adopt a position ideal for pollination success, which is similar to the function of the sterile inflorescence axis in *Babiana ringens* (Iridaceae)^10^. The maintenance of the mutualism between flowers and pollinators demands the precise pollination during nectar foraging by animals. If these animals act as nectar robbers or nectar thieves, getting the rewards but without serving flowers, both male and female fitness of plants will be decreased^24^,^25^. The presence of the TAC significantly promoted the reproductive fitness, which is probably why flowers invest such considerable resource on this unusual structure of the anther crest. The TAC here functions to actively manipulate pollinator’s foraging behavior, which is different from any other previously reported anther appendages that exclusively function to passively adapt for the pollinator’s foraging behaviours. So far as we know, anther appendages in *Roscoea* gingers, *Salvia* (Lamiaceae) and *Torenia* (Scrophulariaceae) function as levers to tilt the anther thus facilitating pollen loading on pollinators^11^,^12^,^26^; in *Globba* gingers, the wing-like anther appendage, extended from the two sides of the anther, has been hypothesized to promote the anther full contact with the pollinator^27^; and anther appendages in other angiosperms play a role in anther dehiscence and pollen dispensing^13^,^28^.

Although the TAC is a male organ derived from the anther, it has functions of both the male and the female. In our study, flowers without the TAC had significantly fewer pollen grains transported out during a single visit by pollinators (Fig. 2B), and produced fewer fruits and seeds than the intact flowers (Fig. 2C). The contribution of TAC on female function is 51.4%, higher than that in male function, which is 40.7%, according to our evaluation. The TAC therefore functions both male and female, with the female function even greater than the male function. The function of the anther appendage in *Z. densissimum* is different from what have been reported in other angiosperm families where the function of anther appendages is restricted to the male. For example, the anther appendage in *Salvia* (Lamiaceae) and *Torenia* (Scrophulariaceae) functions as levers to tilt the anther thus facilitating pollen loading on pollinators, but with little function in female^11^,^12^; anther appendages in Ericaceae, genera of *Viola* and *Hybanthus* (Violaceae), and *Incarvillea* (Bignoniaceae) have a function in anther dehiscence and pollen dispensing^13^,^28^,^29^,^30^,^31^. Unlike other angiosperm species, the filiform styles of gingers are soft and hold between pollen sacs of anthers, and form an anther-stigma cooperation system, which is the characteristic of Zingiberaceae. This is possibly why the anther crest contributes on both the male and the female functions. The anther appendage in *Z. densissimum* may have evolved under selective pressure imposed via the reproductive fitness of both the male and the female. The TAC, derived from the anther connective, is a part of the anther and morphologically male. The TAC will therefore be attributed to resource allocation on the male organ according to sex allocation theory^32^. However, it has both the male and the female roles in function. The contradiction between the morphological gender and the ecological function for TAC indicates that the methodology in sexual allocation theory of hermaphrodites, which partitions floral organs into exclusively male or female^32^,^33^, is not appropriate. The purpose of diverse strategies of resource allocation on sexual organs is to achieve the maximum total fitness (male fitness + female fitness). In doing so, one sexual organ may have the complementary effect on the alternative sexual role within a flower.

Our study indicates the TAC, a contrivance of the anther (male organ), functions to manipulate pollinator’s behaviour, thus enhancing both male and female functions. This study provides a novel example of structure adaptation in which both the male and the female fitnesses are enhanced by resource allocation on a device derived from the male organ, expanding the knowledge of the sexual role of the anther appendage.

## Methods

### Study sites and species

This study was conducted in Lincang, Yunnan province, southwest of China (23°35′ N, 100°04′ E; altitude 1890 m) from 2011 to 2012. Individuals of *Zingiber densissimum* in this wild population grow in a pine forest. In this study, permission was got from the owner of the land to conduct the study on this site. No endangered or protected species were involved.

*Zingiber densissimum* is a perennial herb endemic to southwest Yunnan province in China. The individual generally has one to four conical inflorescences arising from rhizomes, with 10 to 20 flowers per inflorescence and one flower opening on each inflorescence per day. Flowers are opening in the morning and closing in the late evening, with the floral longevity less than one day. The tubular flower has three corolla lobes and six stamens, with four of the stamens petaloid and unfertile, one reduced and only one fertile. The anther is light yellow, with connective extended apically into a tail-like anther crest (Fig. 1A,B). This species is an outcrossing species, and sexual reproduction demands pollination by insects^17^.

### Functions of the TAC

To examine the effect of the TAC removal on the interference on seed provisioning, single flowers from more than 60 individuals were randomly and evenly allocated in two groups: (1) flowers were anther crest-removed and hand self-pollinated, and (2) flowers were intact and hand self-pollinated. All the flowers were bagged during flowering stage. Fruits were collected after one month for seed count. A t-test was used to test the difference on seed production.

To investigate whether the TAC functioned to attract pollinators, 50 individuals were selected. For each individual, two simultaneously opening flowers were labelled as a pair, with the distance of the two flowers less than 15 cm in each pair. Flowers were bagged before anthesis to decrease the difference in nectar reward. The anther crest was removed randomly from one of each pair of flowers, with the other intact as the control. The flower that pollinator first visited was recorded in each pair. To reduce the specific preference of pollinator individuals, floral pairs were set at different patches and/or different days. The difference of pollinator preference between the two groups was analyzed using a chi-square test. In this study, two pollinator species were distinguished during field observation. Given that the two bee species were the effective pollinators of *Z. densissimum*^17^, they were equally treated and summed up during analysis.

To explore the function of the TAC on the manipulation of pollinator’s behaviours, single flowers from more than 60 individuals were randomly and evenly allocated in two groups: (1) the TAC was removed from flowers, and (2) flowers were intact. Flowers of the two groups were then exposed to pollinators. During the process of pollinator visit on each flower, pollinator species and behaviours were observed. Whether the pollinator could push up the anther and touch the stigma was recorded as the effectiveness of each visit. The difference of pollination success of pollinators between the two groups was analyzed using a logistic model.

### Evaluation on the sexual roles of the TAC

To evaluate the effect of the TAC on male fitness, single flowers from more than 60 individuals were randomly selected in 2011 and evenly allocated in two groups: (1) the TAC was removed from flowers, and (2) flowers were intact. Each flower was bagged before anthesis, and permitted one visit by pollinator after anthesis. Pollen grains left on the flower were then collected for pollen number count. A two–way ANOVA was used to test the difference of the remaining pollen between two groups, with factors of treatment, bee species and treatment × bee species considered. The data was under the reciprocally transformed to meet the normality. The average number of pollen grains left after a single visits by pollinators in intact flower group was *P*_*i* ,_ while the number of pollen grains left in the flower group with anther crest removed was *P*_*m*_ . In addition, 30 floral buds were collected for total pollen number (*P*_*t*_) counting. The contribution of TAC on male function (*C*_*m*_) was then evaluated by pollen removal via equation (1):


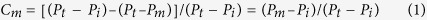


To examine the effect of the TAC on female fitness, single flowers from more than 60 individuals were selected in 2011and allocated in two groups: (1) the TAC was removed from flowers, and (2) flowers were intact. Flowers were manipulated in the early morning, tagged and open pollinated, which allowed flowers to be visited for numerous times during the entire flowering stage. Fruits were collected one month after pollination. The two treatments were repeated in 2012. The difference of fruit set between two groups was analyzed using a logistic model, and the difference of seed number was analyzed using a two-way ANOVA. The average number of seeds in intact flower group was *S*_*i* ,_ while the number of seeds in the flower group with anther crest removed was *S*_*m*_. The contribution of TAC on female fitness (*C*_*f*_) was then evaluated by seed production via equation (2):


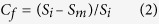


Unlike the evaluation on male function where flowers were permitted only one visit by bees, flowers during the evaluation on female function were open pollinated in the entire flowering and permitted multiple visits by pollinators. The manipulated flowers that were not pollinated at the first visit may get pollinated during following visits by bees, and several effective visits by pollinators would be sufficient for the achievement of female fitness^33^. The estimation on female function of TAC is therefore more conservative than that on male function.

In this study, all the analyses were performed in R 3.02 for windows, and data was expressed as mean ± standard error.

## Additional Information

**How to cite this article**: Fan, Y.-L. and Li, Q.-J. Tail-like anther crest aids pollination by manipulating pollinator's behaviour in a wild ginger. *Sci. Rep.* **6**, 22340; doi: 10.1038/srep22340 (2016).

## Supplementary Material

Supplementary Information

Supplementary Video 1

Supplementary Video 2

## Figures and Tables

**Table 1:** Results of an analysis of deviance (χ^2^) and a two-way ANOVA (F) examining the differences in effectiveness of pollinator visitation and the remaining pollen grains after a visit by bees between the control and the appendage removal group in *Zingiber densissimum.*

source	Effectiveness of visit			Remaining pollen number		
	DF	χ^2^	p	DF	F	P
Treatment	1	27.205	**< 0.001**	1	15.823	**<0.001**
Bee species (BS)	1	0.167	0.6826	1	2.677	0.1074
Treatment ×BS	1	0.387	0.5338	1	2.379	0.1286

**Table 2:** Results of an analysis of deviance (χ^2^) and a two-way ANOVA (F) examining the differences in fruit set and seed number between the control and the appendage removal group in *Zingiber densissimum.*

source	Fruit set			Seed number		
	DF	χ^2^	p	DF	F	p
year	1	0.738	0.3901	1	3.491	0.0642
treatment	1	7.420	**0.0064**	1	45.170	**<0.001**
Year × treatment	1	1.077	0.2994	1	4.672	0.0372
