# Supplementary material for: Tail-like anther crest aids pollination by manipulating pollinator’s behaviour in a wild ginger
Source: Sci Rep. 2016 Mar 1;6:22340. doi: 10.1038/srep22340 (PMC4772108; doi:10.1038/srep22340)
Supplement: Supplementary Information [file srep22340-s1.pdf]

Electronic supplementary Materials for:

Tail-like anther crest aids pollination by manipulating pollinator's behaviour in a wild ginger

Yong-Li Fan [1, 2] and Qing-Jun Li [1, *]

[1]Key Laboratory of Tropical Forest Ecology, Xishuangbanna Tropical Botanical Garden, the Chinese Academy of Sciences, 88# Xuefu Road, Kunming, Yunnan 650223, China. [2]China Forest Exploration and Design Institute of Kunming, Kunming 650216, China.

[*] For correspondence. E-mail qjli@xtbg.ac.cn

Table of content:

1 Supplementary Data……………………………………………………………………………2

1.1 Floral manipulation and pollinator preference ………………………………………………2

1.2 Floral manipulations, pollinator species and pollinator behavior……………………………2

1.3. Floral manipulation, pollinator species and pollen left after a single visit by bees………2

1.4. Floral manipulation, year and seed production (fruit set and seed number)………………2

1.5. The evaluation of the contribution of the anther appendage on male and female, respectively ………………………………………………………………………………………3

2 Video legends ………………………………………………………………………………3

2.1 Supplementary video 1 - Legitimate visit to intact flowers……………………………………3

2.2 Supplementary video 2 - Illegitimate visit to TAC-removed flowers…………………3

1 Supplementary Data

1.1 Floral manipulation and pollinator preference

Treatment	Whether the flower got first visit in each floral pair	Visit number
Intact	Yes	29
	No	21
Appendage removal	Yes	21
	No	29

1.2. Floral manipulations, pollinator species and pollinator behavior

Treatment	Pollinators observed	Pollination effectiveness	Visit number
Intact	Ventral bee	Yes	34
		No	4
	Dorsal bee	Yes	6
		No	1
Appendage removal	Ventral bee	Yes	13
		No	24
	Dorsal bee	Yes	3
		No	3

1.3. Floral manipulation, pollinator species and pollen left after a single visit by bees, with sample size in brackets.

Flower treatment	Pollinator species	Remaining pollen grains (mean ± s.e.)	Total remaining pollen grains (mean ± s.e.)
intact	Ventral bee	6636 ± 562	6541 ± 176 (n=30)
	Dorsal bee	6522 ± 121	
Appendage removal	Ventral bee	8214 ± 342	7453 ± 198 (n=30)
	Dorsal bee	7263 ± 218	

1.4. Floral manipulation, year and seed production (fruit set and seed number).

Year	Treatment	Fruit set	Mean seed number	Sample size
2011	Intact	81.5 %	16.1 ± 0.75	54
	Appendage removal	69.2%	9.22 ± 0.67	52
2012	Intact	83.9 %	11.03 ± 0.85	31
	Appendage removal	54.8 %	9.82 ± 1.15	31

1.5. The evaluation of the contribution of the anther appendage on male and female, respectively.

		2011		2012
	Original pollen grain number	Pollen left after single visited by bees	Seed production	Seed production
Intact Flower	8782 ±162	6541 ± 132	13.13 ± 1.05	10.88 ± 1.08
appendage-removed flower		7453 ± 198	6.38 ± 0.75	5.39 ± 1.09
Contribution of appendage on reproductive fitness		40.7 % (male)	51.41% (female)	50.45% (female)

2 Video legends

2.1 Supplementary video 1 - Legitimate visit to intact flowers

A bee (*Amegilla zonata*, Apidae) probed nectar of an intact flower in upright-down manner and pushed up the TAC with its legs, achieving ventral pollination (legitimate visit). The TAC acted as a handle for pollinators to push up the anther during their nectar-foraging process.

2.2 Supplementary video 2 - Illegitimate visit to TAC-removed flowers

In a TAC-removed flower, a bee (*Amegilla zonata*, Apidae) directly inserted the proboscis into the floral tube from the side of the filament, without touching the stigma and the anther (illegitimate visit).
